# Test-Retest Reliability of PODOSmart^®^ Gait Analysis Insoles

**DOI:** 10.3390/s21227532

**Published:** 2021-11-12

**Authors:** Andreas Loukovitis, Efthymios Ziagkas, Dimitrios Xypolias Zekakos, Alexandros Petrelis, George Grouios

**Affiliations:** 1 Department of Physical Education and Sport Science, Aristotle University of Thessaloniki, 57001 Thessaloniki, Greece; loukovit@phed.auth.gr (A.L.); eziagkas@phed.auth.gr (E.Z.); 2 Digitsole SAS, 54000 Nancy, France; d.zekakos@group-epsilon.com (D.X.Z.); apetrelis@live.com (A.P.)

**Keywords:** gait analysis, test-retest reliability, repeatability, spatiotemporal gait characteristics, insoles, PODOSmart^®^

## Abstract

It is recognized that gait analysis is a powerful tool used to capture human locomotion and quantify the related parameters. PODOSmart^®^ insoles have been designed to provide accurate measurements for gait analysis. PODOSmart^®^ insoles are lightweight, slim and cost-effective. A recent publication presented the characteristics and data concerning the validity of PODOSmart^®^ insoles in gait analysis. In literature, there is still no evidence about the repeatability of PODOSmart^®^ gait analysis system. Such evidence is essential in order to use this device in both research and clinical settings. The aim of the present study was to assess the repeatability of PODOSmart^®^ system. In this context, it was hypothesized that the parameters of gait analysis captured by PODOSmart^®^ would be repeatable. In a sample consisting of 22 healthy male adults, participants performed two walking trials on a six-meter walkway. The ICC values for 28 gait variables provided by PODOSmart^®^ indicated good to excellent test-retest reliability, ranging from 0.802 to 0.997. The present findings confirm that PODOSmart^®^ gait analysis insoles present excellent repeatability in gait analysis parameters. These results offer additional evidence regarding the reliability of this gait analysis tool.

## 1. Introduction

**Figure 1:**
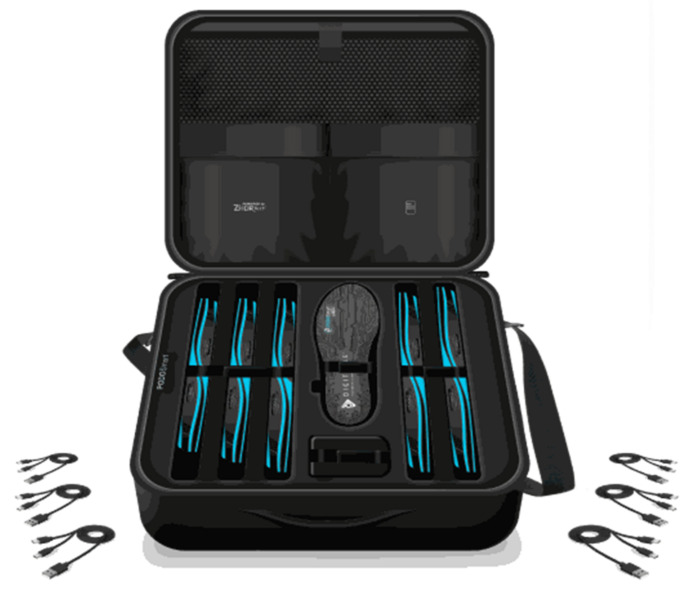
PODOSmart^®^ insoles gait analysis kit.

Gait analysis has been recognized as a standard and powerful tool used to capture human locomotion and quantify the related parameters [1]. It is used to gain insight into the spatial and temporal gait characteristics [2]. In order to reach this objective, new smart insoles, PODOSmart^®^ (Digitsole SAS), have been developed. PODOSmart^®^ insoles have been designed to provide accurate measurements for gait analysis (Figure 1). Key advantages of PODOSmart^®^ include being lightweight, having a slim design and being cost-effective. A recent publication presented the characteristics of these insoles and the validation study of PODOSmart^®^ insoles’ calculated parameters compared to the gold standard Vicon system (Vicon MX, Oxford Metrics, Oxford, UK) [3]. It is important to highlight that the PODOSmart^®^ system accurately captures data wherever gait takes place. This means that, instead of being limited to the laboratory, PODOSmart^®^ can record data in all environments and fields where gait usually takes place. 

When developing a new device such as PODOSmart^®^, it is crucial to measure its repeatability. Unless repeatability is checked, emerging data are of no value. As such, the importance of repeatability measurement cannot be underestimated as it determines the device’s reliability. Low repeatability leads to a negative impact on the device’s quality. On the other hand, the affirmation of high repeatability means that the device can be used appropriately. Therefore, precise measurements are a prerequisite for the quality of a device and ensures its repeatability. 

Repeatability or test-retest reliability indicates the agreement between multiple assessments of the same measurement under the same conditions [4]. It is commonly assessed using the calculation of intraclass correlation between the first measurement and subsequent measurement [5]. With respect to the motion system, repeatability can be considered more important than accuracy [6]. Hence, the evaluation of repeatability is of great importance when developing new measures for gait analysis. The confirmation of repeatability means that the administrations of the measure at two distinct occasions result in consistent measurements. In turn, it implies the precision of measurements [7]. 

With respect to insole-based systems, several researchers have been focused on the validation of these state-of-the-art technology systems. For instance, the commonly used Pedar system has demonstrated good repeatability and validity [8,9]. In another study, the validity and reliability of the pressure-measure insoles “OpenGo” were investigated, and it was found that the system is appropriate for measuring kinetic and spatiotemporal gait parameters [10]. More specifically, for all the parameters measured, an intra-class correlation was >0.796 for validation while correlation was >0.994 for reliability. Later, Stöggl and Martiner (2017) [11] added to the work of Braun et al. (2015) [10] by concluding that “OpenGo” can evaluate gait parameters during different types of motion such as running or jumping. In addition, Oerbekke et al. (2017) [12] confirmed that “OpenGo” is a valid and reliable device for measuring gait parameters during walking. By adopting similar research methods, other researchers tested the repeatability of “Loadsole” insole system and found that it can be used for assessing ground-based kinetics [13]. Similarly, “Medilogic” and “Tekscan” devices displayed good repeatability between measurements [14]. In addition, previous research efforts confirmed the repeatability of other insole systems such as BioFoot^®^ [15] and Pedar-X system^®^ [16]. Concluding in literature, the repeatability of insole devices have been investigated through protocols with varying methodologies. In this context, it should be noted that in validation studies, the methodology approach used is a major concern. A suitable approach fulfils the aims of the study and reaches reliable conclusions about repeatability. 

In literature, there is still no evidence about the repeatability of PODOSmart^®^. Such evidence is essential in order to use these devices in both research and clinical settings. Thus, the present study aimed to assess the repeatability of PODOSmart^®^ system. In this context, it was expected that the parameters of gait analysis captured by PODOSmart^®^ would be repeatable.

## 2. Materials and Methods

### 2.1. Samples

As it is well documented in recent bibliography that age and gender play a significant role on human gait parameters [17,18], this study sample consisted of 22 healthy male adults with ages ranging from 20 to 51 years (mean age was 34.27 ± 7.47 years). Their mean height was 1.73 ± 6.65 m (from 1.60 to 1.84 m range), and their mean weight was 74.90 ± 6.70 kg (ranging from 62 to 90 kg). To qualify for the study, participants could not have had any known gait abnormalities. Participants were recruited from the Department of Physical Education and Sport Sciences of the Aristotle University of Thessaloniki. Their participation was voluntary.

### 2.2. Ethical Considerations

The present study is based on the ethical guidelines of the Research and Ethics Committee of Aristotle University of Thessaloniki in Greece (Approval number 76/2021). Researchers asked participants to give written consent before being part of this study. In addition, human and ethical standards according to the Declaration of Helsinki were followed.

### 2.3. Experimental Protocol

**Figure 2:**
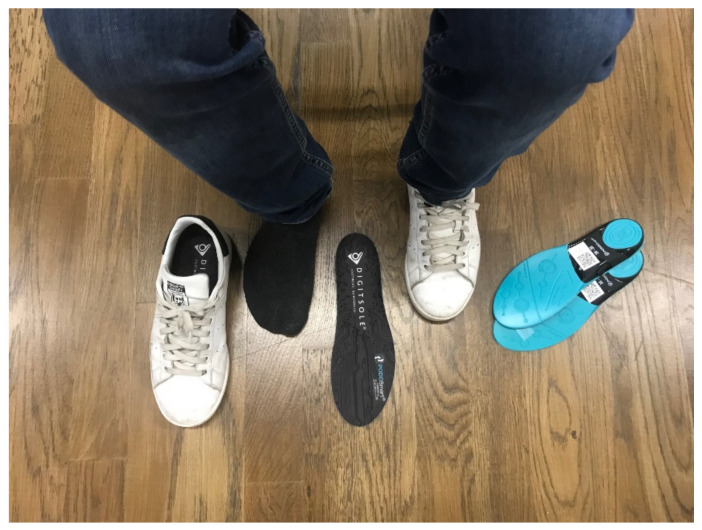
Subject preparation and PODOSmart^®^ insoles fitting.

Data of the spatial and temporal gait parameters were recorded. PODOSmart^®^ insoles were installed inside participants’ shoes (Figure 2). Participants were requested to walk on a 6-m walkway located within the Motor Control and Adapted Physical Activity laboratory at the Department of Physical Education and Sport Science of the Aristotle University of Thessaloniki. Each participant performed two walking trials at their preferred walking speed. During walking acquisitions, turns and U-turns were allowed. The interval between the two trials was 20 min. Participants were informed that they could perform practice walks to get acquainted with the experimental procedure’s insoles. Additionally, they were asked to wear sport shoes, t-shirts and shorts in order to feel comfortable. During the measurements, participants were instructed to walk at a self-selected speed. 

### 2.4. Instruments

#### PODOSmart^®^ Movement Analysis

**Figure 3:**
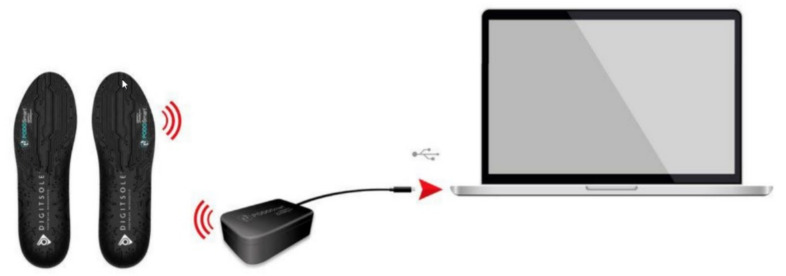
Data recording and transfer procedure.

PODOSmart^®^ can be used as a valuable tool in the assessment of gait parameters. This tool has been presented in detail previously [3]. It captures data while walking or running in real-life activities. PODOSmart^®^ includes six pairs of insoles connected to a mobile application and an easy-to-use software. Walking steps, running strides and foot orientations in space are measured by an inertial platform located in PODOSmart^®^ insoles. Each Podosmart^®^ insole has an inertial platform that records the movements and orientations of each foot in space. At the end of each acquisition, data from insoles are transferred at the PodoStation (wireless connection box) and them processed by PODOSmart^®^ artificial intelligence algorithms. The artificial intelligence algorithms of PODOSmart^®^ insoles process these measurements and estimate the spatial, temporal, kinematic and biomarker parameters (Figure 3). These parameters are then displayed in a proprietary interface (Table 1). PODOSmart^®^, as a state-of-the-art smart insole, weighs 66 g and is available in different sizes. They allow active use for continuous 33 h since they are rechargeable via USB. The above characteristics contribute sο that the PODOSmart^®^ system provides immediate biofeedback.

### 2.5. Data Analysis

Data and statistical analysis were performed using the Statistical Package for Social Sciences, SPSS Version 25. The examination of test and retest reliability was carried out using the Intraclass Correlation Coefficient. Intraclass Correlation Coefficient (or ICC) is widely used for two or more data sets and has the advantage that it does not overestimate relationships for small samples. A two-way mixed-effects model was used to calculate the ICC with measures of consistency. The two-way mixed-effects model is proposed to be used only in cases where the selected raters are the only raters of interest [19]. ICC values less than 0.5 indicate poor reliability, values between 0.5 and 0.75 are indicative of moderate reliability, values between 0.75 and 0.9 indicate good reliability and values greater than 0.90 indicate excellent reliability [19]. Furthermore, the Standard Error of Measurement (SEM), the Minimal Detectable Change at 95% confident (MDC_95_) and the Minimal Detectable Change as a percentage (MDC%) were also calculated [20,21]. The *p*-value was set at the level of 0.05.

## 3. Results

Descriptive statistics including means and standard deviations of all examined gait variables, in both the first and second gait analysis, performed using PODOSmart^®^ insoles are presented in Table 2. 

The ICC values for 28 gait variables provided by PODOSmart^®^ indicated good to excellent test-retest reliability, ranging from 0.802 (digitigrade in milliseconds of the right foot) to 0.997 (contact time in milliseconds of left foot). Regarding bipedal gait variables, excellent test-retest reliability was found between walking speed measurements. The average measure ICC in walking speed was 0.924 with a 95% confidence interval from 0.825 to 0.968 (F(21,21) = 25.176, *p* = 0.000). Excellent test-retest reliability was also found between cadence measurements. The average measure ICC in cadence was 0.932 with a 95% confidence interval from 0.844 to 0.971 (F(21,21) = 28.552, *p* = 0.000). 

Concerning temporal gait characteristics, for contact time on the left foot, the average measure ICC was 0.997, while for the right foot, the average measure ICC was 0.989. In swing time for the left foot, the average measure ICC was 0.960, and for the right foot, the average measure ICC was 0.906. In the taligrade of the left foot, the average measure ICC was 0.979, while for the right foot, the average measure ICC was 0.973. In the plantigrade of the left foot, the average measure ICC was 0.919, and in the plantigrade of the right foot, the average measure ICC was 0.839. The last characterized variable of temporal gait was digitigrade, in which the left foot, the average measure ICC was 0.842, and the right foot, the average measure ICC was 0.802.

Regarding spatial gait characteristics in foot progression angle, the average measure ICC was 0.975 for the left foot and 0.973 for the right foot. For clearance of the left foot, ICC was 0.816, while for the right foot it was 0.811. For steppage of the left foot, the average measure ICC was 0.939 and 0.900 for the right foot. In stride length of the left foot, the average measure ICC was 0.845, while for the right foot, it was 0.907. 

Regarding angles at initial contact to toe-off phases, in heel strike of the left foot, the average measure ICC was 0.952, while for the right foot, it was 0.930. In the toe strike of the left foot, ICC was 0.916, and for the right foot, it was 0.917. In heel off for the left foot, the average measure ICC was 0.914, while for the right foot the ICC was 0.897. Finally, in toe-off for the left foot, the average measure ICC was 0.848, while for the right foot, ICC was 0.845. Intraclass correlation coefficient for each variable as well as the lower bound, upper bound and the significance are presented in Table 3.

## 4. Discussion

This paper aimed to examine the test-retest reliability of a newly introduced inertial measurement unit (IMU) based measuring insole device, PODOSmart^®^ for gait analysis. Gait analysis data were collected from 22 healthy male subjects. Each participant performed two walking trials and for the test-retest reliability, we used ICC with a two-way mixed-effects model with measures for consistency between the two walking acquisitions. 

Gait analysis requires accurate measurements of gait parameters [22]. Over the years, advances in technology have led to the development of new technologies for gait analysis. In order to measure gait variables, multiple technologies have been developed, including 3D video capturing or sensors-based devices [23,24]. Although 3D motion video capturing technology offers high accuracy measurements, the cost of using this technology is prohibitive [25]. New low-cost devices have been developed to fill the gap in this sector [23]. However, the study of their validity and accuracy is still ongoing [2,26,27].

The results of this paper indicate that PODOSmart^®^ insoles demonstrated a good to excellent test-retest reliability. ICC ranged from 0.802 to 0.997, with 19 of 28 variables presenting ICC ≥ 0.90, and only nine variables presenting ICC between 0.80 and 0.90. More specifically, in spatiotemporal gait variables (walking speed and cadence), the ICC showed excellent test-retest reliability (ICC = 0.924 and ICC = 0.932, respectively). Concerning spatial gait variables such as clearance and stride length for each foot, the present findings indicated good to excellent test-retest reliability (ICC ranging from 0.811 to 0.924). With regards to temporal gait characteristic, results showed good to excellent test-retest reliability (ICC ranging from 0.802 for digitigrade of the right foot to 0.997 for contact time of the left foot). Respecting gait angles, ICC ranged from 0.845 to 0.975 showing good to excellent test-retest reliability (ICC = 0.845 for the right foot toe and ICC = 0.975 for foot progression angle of the left foot). 

Current results are coherent with other studies examining the test and retest reliability of insole-based sensors [8,9,10,11,12,14,15,16,23,28,29]. However, the differences in the specific type of sensors used [16,30] and methodological limitations such as various spatiotemporal gait variables [13,28], several motor tasks [27,30] or the use of non-typical populations, do not allow direct comparisons of the results. 

Regarding the specific type of sensors used, Godi et al. in their study in 2014, used a plantar pressure system in order to assess gait along linear and curved trajectories [16]. In the same year, Castro et al. (2014) reported that the WalkinSense device had good-to-excellent levels of accuracy and repeatability for plantar pressure variables [29]. In 2021, Barratt et al. examined test-retest reliability of Moticon pressure sensor insoles measuring plantar pressure and reaction force, and they found moderate to strong test-retest reliability [30]. Concerning methodological limitations, Healy et al. in 2012, used insoles containing plantar pressures sensors and found differences concerning the repeatability between a day-to-day plantar pressure measurement [28]. Price et al. in 2016, examined the test-retest reliability of three in-shoe pressure measurement devices (Medilogic, Pedar and Tekscan) and reported that average and peak pressures demonstrated high between-day repeatability for all three systems and each insole size (ICC ≥ 0.859) [14]. Furthermore, Peebles et al. in 2018, examined the validity and repeatability of the single-sensor Loadsol insoles during single-hop and stop-jump landing and their findings indicated that repeatability ICC were moderate to excellent (ranging from 0.616 to 0.928) [13]. With regard to several motor tasks, Antwi-Afari et al. in 2020, used a wearable insole pressure system aiming to examine five gait parameters (stride time, stride length, swing time, stance time and single support time) in laboratory fall-risk events and found test-retest reliability ICC = 0.910 [27]. Accordingly in the study of Barrat et al. in 2021, Moticon pressure sensor insoles had been used to measure plantar pressure and reaction force during ergometer rowing and reported moderate to strong test-retest reliability (ICC ranging from 0.57 to 0.92) for mean and peak plantar pressure and reaction force [30]. With respect to the populations participating in previous studies, Farid et al. in 2021, in a sample of 29 stoke patients using FeetMe^®^ monitor-connected insoles, reported test-retest and inter-rater ICCs > 0.73 for walking speed, stride length, cadence, stance and swing duration [31].

Comprehensively, the present study indicated that PODOSmart^®^ insoles present good to excellent repeatability in all gait analysis variables as offered by the PODOSmart^®^ interface. The present findings are essential in order to expand the use of PODOSmart^®^ insoles in both research and clinical settings.

## 5. Conclusions

In conclusion, the results of this study confirm that PODOSmart^®^ gait analysis insoles present excellent repeatability in gait analysis parameters. These results offer additional evidence regarding the reliability of this gait analysis tool. The present findings may be used in order to develop a personalized system using machine learning algorithms. This opportunity will allow further research studies to test the reliability of PODOSmart^®^ gait analysis insoles in non-typical gait, such as neurologic or orthopedic conditions or special populations. 

## Figures and Tables

**Table 1:** Gait analysis variables provided by PODOSmart^®^.

Spatiotemporal Variables	Spatial Variables	Temporal Variables	Angles
Walking speed	Clearance	Contact time	Heel strike
Cadence	Stride length	Flying time	Toe strike
		Taligrade	Heel off
		Plantigrade	Toe off
		Digitigrade	Foot progression angle
			Steppage

**Table 2:** Descriptive statistics concerning PODOSmart^®^ gait variables between the two measurements.

Gait Variables	1st Measurement					2nd Measurement				
	M	SD	SEM	MDC_95_	MDC%	M	SD	SEM	MDC_95_	MDC%
Contact time (Left foot)	798.8	92.22	19.66	54.49	6.82	798.0	93.94	20.03	55.52	6.96
Contact time (Right foot)	806.6	96.94	20.67	57.29	7.10	806.3	93.97	20.04	55.55	6.89
Flying time (Left foot)	506.9	40.18	8.57	23.75	4.69	512.6	44.56	9.50	26.33	5.14
Flying time (Right foot)	522.5	46.96	10.01	27.75	5.31	529.7	47.45	10.12	28.05	5.30
Taligrade (Left foot)	162.6	81.95	17.47	48.42	29.78	158.9	83.99	17.91	49.64	31.24
Taligrade (Right foot)	147.6	73.32	15.63	43.32	29.35	146.8	71.07	15.15	41.99	28.61
Plantigrade (Left foot)	375.3	87.51	18.66	51.72	13.78	370.9	94.86	20.22	56.05	15.11
Plantigrade (Right foot)	391.0	94.87	20.23	56.07	14.34	393.2	105.11	22.41	62.12	15.80
Digitigrade (Left foot)	260.5	25.03	5.34	14.80	5.68	262.7	25.45	5.43	15.05	5.73
Digitigrade (Right foot)	264.6	27.01	5.76	15.97	6.03	264.5	34.05	7.26	20.12	7.61
Food progression angle (Left foot)	8.7	9.50	2.03	5.63	64.68	7.7	9.62	2.05	5.68	73.80
Food progression angle (Right foot)	8.2	7.50	1.60	4.43	54.09	8.4	7.34	1.57	4.35	51.81
Clearance (Left foot)	1.6	0.56	0.12	0.33	20.79	1.6	0.66	0.14	0.39	24.25
Clearance (Right foot)	1.7	1.09	0.23	0.64	37.50	1.8	1.15	0.25	0.69	38.50
Steppage (Left foot)	18.2	6.24	1.33	3.69	20.26	17.7	5.80	1.24	3.44	19.42
Steppage (Right foot)	15.2	5.42	1.16	3.22	21.15	16.3	5.92	1.26	3.49	21.43
Walking speed	3.1	0.71	0.15	0.42	13.41	3.1	0.65	0.14	0.39	12.52
Stride length (Left foot)	112.9	11.12	2.37	6.57	5.82	112.5	10.43	2.22	6.15	5.47
Stride length (Right foot)	117.5	12.80	2.73	7.57	6.44	117.5	12.55	2.68	7.43	6.32
Cadence	91.4	11.13	2.37	6.57	7.19	90.5	10.76	2.29	6.35	7.01
Heel strike (Left foot)	−12.8	5.05	1.08	2.99	23.39	−12.7	5.10	1.09	3.02	23.79
Heel strike (Right foot)	−15.1	5.20	1.11	3.08	20.38	−15.4	5.19	1.11	3.08	19.98
Toe strike (Left foot)	−7.0	3.10	0.66	1.83	26.13	−6.5	2.76	0.59	1.64	25.16
Toe strike (Right foot)	−7.4	3.60	0.77	2.13	28.84	−7.9	3.82	0.82	2.27	28.77
Heel off (Left foot)	−6.2	2.27	0.48	1.33	21.46	−6.2	2.32	0.49	1.36	21.91
Heel off (Right foot)	−6.5	3.59	0.77	2.13	32.84	−6.3	3.00	0.64	1.77	28.16
Toe off (Left foot)	−8.1	3.85	0.82	2.27	28.06	−7.9	3.61	0.77	2.13	27.02
Toe off (Right foot)	−5.4	3.43	0.73	2.02	37.47	−5.0	2.99	0.64	1.77	35.48

**Table 3:** Intraclass Correlation Coefficient values for each variable as measured using PODOSmart^®^ gait analysis insoles.

Variable	Degree of Reliability	Average Intraclass Correlation	Lower Bound	Upper Bound	Sig.
Contact time (ms) (L)	Excellent	0.997	0.997	0.999	0.000
Contact time (ms) (R)	Excellent	0.989	0.974	995	0.000
Swing time (ms) (L)	Excellent	0.960	0.906	0.983	0.000
Swing time (ms) (R)	Excellent	0.906	0.787	0.960	0.000
Taligrade (ms) (L)	Excellent	0.979	0.951	0.991	0.000
Taligrade (ms)(R)	Excellent	0.973	0.937	0.989	0.000
Plantigrade (ms) (L)	Excellent	0.919	0.816	0.966	0.000
Plantigrade (ms) (R)	Good	0.839	0.652	0.930	0.000
Digitigrade (ms) (L)	Good	0.842	0.658	0.931	0.000
Digitigrade (ms) (R)	Good	0.802	0.581	0.913	0.000
Foot progression angle (°) (L)	Excellent	0.975	0.941	0.990	0.000
Foot progression angle (°) (R)	Excellent	0.973	0.936	0.989	0.000
Clearance (cm) (L)	Good	0.816	0.607	0.919	0.000
Clearance (cm) (R)	Good	0.811	0.599	0.917	0.000
Steppage (°) (L)	Excellent	0.939	0.859	0.994	0.000
Steppage (°) (R)	Excellent	0.900	0.755	0.957	0.000
Walking speed (km/h)	Excellent	0.924	0.825	0.968	0.000
Stride length (cm) (L)	Good	0.845	0.663	0.933	0.000
Stride length (cm) (R)	Excellent	0.907	0.760	0.960	0.000
Cadence (steps/min)	Excellent	0.932	0.844	0.971	0.000
Heel strike (°) (L)	Excellent	0.952	0.889	0.980	0.000
Heel strike (°) (R)	Excellent	0.930	0.838	0.970	0.000
Toe strike (°) (L)	Excellent	0.916	0.809	0.964	0.000
Toe strike (°) (R)	Excellent	0.917	0.811	0.965	0.000
Heel off (°) (L)	Excellent	0.914	0.804	0.963	0.000
Heel off (°) (R)	Good	0.897	0.769	0.956	0.000
Toe off (°) (L)	Good	0.848	0.668	0.934	0.000
Toe off (°) (R)	Good	0.845	0.663	0.933	0.000

## Data Availability

The data presented in this study are available on request from the corresponding author. The data are not publicly available due to privacy and ethical restrictions.
